# Singular Hallucination

**Published:** 1853-10

**Authors:** 


*Singular Hallucination.*

The case of Alexander Lewis, who was tried and acquitted for murder at the present (Sept.) Term of the Court of Common Pleas of Chicago.

We are indebted to John A. Thompson, Esq., of this city, counsel for the prisoner, for the following facts relative to the trial and the history of Lewis.

The crime was committed at a grove near Ridgeville, in this county, last spring, and from the enormity of the deed and the singular hallucination that prompted it, we should consider it an oversight if not laid before the public.

The deceased, a poor negro, and the prisoner—total strangers to each other, while pursuing the lake shore road leading from this city to Milwaukie, on foot, accidentally fell into company—neither having any money, except a dime, which the deceased paid out for whiskey at a grocery by the road side, and of which both drank. They journeyed on together, and a short time after dark arrived at a grove in Ridgeville where they kindled a fire and laid down upon the ground to sleep—the negro with his bundle under his head. The prisoner arose in the night, and while the deceased was asleep, took from a pile of wood near by a large oaken bludgeon, and with it struck the deceased three or four blows upon the head, breaking his skull in several places, and scattering his brains upon the ground. The prisoner pursued his way through Milwaukie and thence to Walworth Co., Wisconsin, where he voluntarily confessed the crime, saying God commanded him to commit it, and surrendered himself into custody. He was subsequently brought to the jail of this city, where he again confessed the crime and described the state of mind that prompted him to do it. The body of the negro was found the next morning in the grove, his budget and contents undisturbed, by his head—the bludgeon stained with blood by his side, and the fire still burning,

Upon the trial there was no controversy as to the act of homicide; the counsel for the prisoner having admitted it, contended and satisfactorily established to the jury and the medical witnesses in attendance, that the prisoner was not responsible for his acts by reason of insanity.

The following facts adduced upon his defence, and which were clearly made out by the proof, and upon which the jury rendered a verdict of not guilty upon their seats, show one of the most frightful cases of mania that ever occurred, and which trial adds another to the many conclusive arguments, showing the inefficiency of the legal "tests of the knowledge of right and wrong," as applied by English and American judges to estimate the degrees of criminal responsibility.

The prisoner, born of religious and humble parents, in eastern New York, was, until the age of about 18 years, remarkable for his amiable and quiet disposition—laboring steadily in the field—beloved and honored by all who knew him—at about that period he, together with his father, became zealous Millerites, both becoming *impressed* with the belief that they were to be taken bodily to heaven—spending most of their time in fasting and prayer. Religious excitement was in his case the pre-existing cause of insanity—the cause that levelled every faculty that raised him above the brute.

After the explosion of Millerism, the family emigrated to Walworth County, Wisconsin, where the prisoner, removed from scenes of excitement for about two years, "was himself again," attending generally to his labors, with but occasional wanderings, until the death of his father, which affected him very materially—he becoming affected to such an extent as to preclude the hope of recovery. During the whole period of his sickness, which lasted nearly a year, he was deranged, talking continually of visions, of himself being possessed of a devil, and frequently remarking that he *must* go direct to hell. Partially recovering (far better that he had then died) he arose from his bed of sickness more deeply afflicted with the awful malady of insanity. At first he believed that property was unequally and sinfully distributed, and that it was his bounden duty to steal from the rich and give to the poor. With this idea he stole some clothing from a store in Walworth County to give to a pauper at that place—confessed—and was put in jail. While in jail he was continually preaching and praying, and at various times fasted from three to five days. Subsequently in jail he became dangerous, tried to kill his keeper, and was chained. When told he could be released, he at first refused to leave, finally said he would see, and engaged in prayer—afterwards said he had permission on condition that he would do no harm for three weeks. He was taken from jail to the house of his uncle near by, and during the first night he arose from his bed, and approached the room where his uncle and aunt were sleeping, saying in a firm and loud voice "now uncle I *must* kill you, it is my command from God." His uncle sprung on him and succeeded in holding him until the household were aroused—a light was brought in, and the maniac's eyes seemed fixed on some wood by the stove. The uncle went to remove it—the prisoner sprung, seized a stick and aimed a blow at his head, which must have proved fatal, had not the mother of the prisoner interposed and received the blow upon her arm, which left a frightful wound and fractured the bone. The prisoner was again immediately taken to jail, and cold weather having set in, (for he was always less deranged in cold weather), he seemingly recovered, was set free and came to Chicago where his brother resided. He was induced to go to work, which he occasionally did through the winter. In the spring (1853) his insanity returned—he attended religious meetings—talked of visions—attempted to induce people to go down to the lake and hear him preach—fasted and prayed as before—attempted to kill his brother in obedience to the command of God—was restless at night—would leave his bed and talk for hours to himself. Finally he became *impressed* with the hallucination, *as of a reality*, that he was *Abraham*, and must offer up some human sacrifice—that it was a necessity he *could* not escape. In such a state of mind he left the city and accidentally met his unfortunate companion, and at Ridgeville grove, perhaps the Mount Moriah of his *crazed* fancy, by the fire with his own hand kindled, and still burning at the altar—with *no* lamb furnished for the offering, he sacrificed his human victim in obedience to imperative duty.

When arraigned and required to plead to the indictment, he said that 'God would defend him;" and when asked if he wanted counsel, his reply was—"God will defend me." When Mr. Thompson, his counsel, attempted to gather from him facts upon which to base an application for continuance, his reply was, that God had told him not to hold any conversation with him; and during the trial he arose from the box, and fixing his eyes upon the Judge, remarked, that God was his judge—that he, the Judge, had better secure a judge to sit upon his own case, than to try him (the prisoner). At all times he *persisted* that he was not insane—he knew that death was the penalty of our laws for murder, yet, he believed himself above human laws—he knew the significance of the Court, the Counsel, and the Jury—and reflected with scorn upon their fancied imbecility—he was the appointed of Heaven for the accomplishment of a great good—was and is rational on subjects, save those connected with his mania—never has attempted suicide—believes himself to be immortal, and, firm as Atlas, insists on his perfect sanity. No—

> " He would not die
> And sanction with self slaughter the dull lie—
> That * * * * * * with the brand of shame
> Stamped madness deep into his memory
> And woo compassion to a blighted name."

Such is a brief statement of the facts of the trial and history of one of the most dangerous and perfect cases of insanity within our experience or reading, and to the indefatigable labors of his counsel in obtaining the witnesses from distant states, a volunteer in his behalf with not so much remuneration even as the poor maniac's sympathy, and in preparing his defence, is due the honor of his acquittal. We also learn that Mr. Thompson has instituted proceedings and obtained an order for his removal to the Lunatic Hospital of this State.

